# Cloning and Characterization of *TpNRAMP3*, a Metal Transporter From Polish Wheat (*Triticum polonicum* L.)

**DOI:** 10.3389/fpls.2018.01354

**Published:** 2018-09-20

**Authors:** Fan Peng, Chao Wang, Yiran Cheng, Houyang Kang, Xing Fan, Lina Sha, Haiqin Zhang, Jian Zeng, Yonghong Zhou, Yi Wang

**Affiliations:** ^1^Triticeae Research Institute, Sichuan Agricultural University, Chengdu, China; ^2^Joint International Research Laboratory of Crop Resources and Genetic Improvement, Sichuan Agricultural University, Chengdu, China; ^3^College of Resources, Sichuan Agricultural University, Chengdu, China

**Keywords:** wheat, NRAMP, metal, plasma membrane, transport, accumulation

## Abstract

Essential transition metals and non-essential metals often co-exist in arable soils. In plants, some transition metal transporters, such as the natural resistance-associated macrophage proteins (NRAMPs), poorly selectively transport metals with similar chemical properties whether they are essential or non-essential. In this study, a member of the NRAMP transporter family, TpNRAMP3, was identified from dwarf Polish wheat (*Triticum polonicum* L.). *TpNRAMP3* encodes a plasma membrane-localized protein and was highly expressed in leaf blades and roots at the jointing and booting stages, and in the first nodes at the grain filling stage. Expression of *TpNRAMP3* increased sensitivity to Cd and Co, but not Zn, and increased the Cd and Co concentrations in yeast. *TpNRAMP3* expression in *Arabidopsis* increased concentrations of Cd, Co, and Mn, but not Fe or Zn, in roots, shoots, and whole plant. However, TpNRAMP3 did not affect translocation of Cd, Co, or Mn from roots to shoots. These results suggest that TpNRAMP3 is a transporter for Cd, Co, and Mn accumulation, but not for Fe or Zn. However, Cd and Co are non-essential toxic metals; selective genetic manipulation of *TpNRAMP3* will help breed low Cd- and Co-accumulating cultivars.

## Introduction

Essential transition metals, such as Fe, Zn, Mn, and Cu, are cofactors of numerous plant proteins and enzymes; therefore, they are required for plant growth and development (Thomine et al., 2000; Lin and Aarts, 2012; Yamaji et al., 2013). Conversely, non-essential metals, such as Cd, Co, and Pb, are highly toxic metals that damage the photosynthetic apparatus, affect respiratory and nitrogen metabolism, and alter the balance of water and nutrient uptake, ultimately inhibiting plant growth and development (Herbette et al., 2006; Balen et al., 2011; Lange et al., 2017). Essential transition metals and non-essential metals often co-exist in arable soils. Therefore, plants need to absorb essential metals to ensure the proper homeostasis of nutrient elements, while excluding non-essential metals to prevent damage (Lahner et al., 2003; Xiao et al., 2008; Lin and Aarts, 2012). However, some transition metal transporters, such as natural resistance-associated macrophage proteins (NRAMPs) and iron-regulated transporters (IRTs), poorly selectively transport metals with similar chemical properties whether they are essential or non-essential (Nevo and Nelson, 2006; Sasaki et al., 2012; Barberon et al., 2014; Pottier et al., 2015).

To date, numerous plant NRAMPs have been characterized from different plant species (Thomine et al., 2000; Nevo and Nelson, 2006; Sasaki et al., 2012; Tejada-Jiménez et al., 2015; Qin et al., 2017; Gao et al., 2018). For *Arabidopsis*, five of six *NRAMP* genes have been functionally identified. Among the identified AtNRAMPs, AtNRAMP1, AtNRAMP3, and AtNRAMP4 all transport Fe, Mn, and Cd (Curie et al., 2000; Thomine et al., 2000, 2003; Lanquar et al., 2005, 2010; Cailliatte et al., 2010; Pottier et al., 2015). AtNRAMP6 also transports Cd, but not Fe or Mn (Cailliatte et al., 2009). Additionally, AtNRAMP3, AtNRAMP4, and AtNRAMP6 are intracellular metal transporters for Cd, Fe, and Mn sequestration and distribution, but not for their uptake (Lanquar et al., 2005, 2010; Cailliatte et al., 2009; Pottier et al., 2015). Only AtNRAMP1 functions in metal uptake of Cd, Fe, and Mn (Curie et al., 2000; Thomine et al., 2000, 2003; Lanquar et al., 2005, 2010; Cailliatte et al., 2009).

In rice, seven *NRAMP* genes have been annotated; five of which have also been functionally characterized. Although the characterized OsNRAMPs are localized at the plasma membrane, they exhibit different metal transport properties (Curie et al., 2000; Xia et al., 2010; Takahashi et al., 2011; Ishimaru et al., 2012; Sasaki et al., 2012; Yamaji et al., 2013; Yang et al., 2013; Li et al., 2014; Tiwari et al., 2014; Peris-Peris et al., 2017). Among them, three OsNRAMPs including OsNRAMP1, OsNRAMP4, and OsNRAMP5 function to take up metals. OsNRAMP1 transports Cd, Fe, As, and Mn (Curie et al., 2000; Takahashi et al., 2011; Tiwari et al., 2014), OsNRAMP4 only absorbs Al, not Fe, Mn or Cd (Xia et al., 2010; Li et al., 2014), and OsNRAMP5 transports Mn, Cd, and Fe (Ishimaru et al., 2012; Sasaki et al., 2012; Takahashi et al., 2014). Although OsNRAMP6 transports Fe and Mn but not Cd or As in yeast (Peris-Peris et al., 2017), it has not been confirmed whether it functions in metal uptake or intracellular transport. Additionally, OsNRAMP3 transports Mn for Mn distribution and remobilization, but not Fe, Cd, or Zn (Yamaji et al., 2013; Yang et al., 2013).

Some *NRAMP* genes have also been identified from other plant species, such as *GmNRAMPs* from soybean (Kaiser et al., 2003; Qin et al., 2017), *MbNRAMP1* from *Malus baccata* (Xiao et al., 2008), *NtNRAMP5* from tobacco (Tang et al., 2017), *TjNRAMP4* from *Thlaspi japonicum* (Mizuno et al., 2005), and *MtNRAMP1* from *Medicago truncatula* (Tejada-Jiménez et al., 2015). Most of their metal transport properties were functionally identified in yeast; only MtNRAMP1 was characterized as a Fe uptake protein in *M. truncatula* (Tejada-Jiménez et al., 2015). However, there is no information on *NRAMP* genes from wheat.

In the present study, a *NRAMP* gene, named *TpNRAMP3*, was isolated from dwarf Polish wheat (DPW, 2*n* = 4*x* = 28, AABB, *Triticum polonicum* L.) that its seedling exhibit high tolerance to Cd and Zn (Wang et al., 2017). Its homologous gene, rice *OsNRAMP3*, encoded a plasma membrane-localized protein, functions as a switch in response to environmental Mn changes, but does not transport Cd, Fe, and Zn (Yamaji et al., 2013; Yang et al., 2013). These results indicate that OsNRAMP3, as a Mn-specific transporter, only mediates Mn distribution in rice. Meanwhile, NRAMPs in different species and different NRAMPs in the same species exhibit different functions for transport of different substrates. Thus, we hypothesized that the function of TpNRAMP3 would differ from those of reported NRAMPs from other species. To test this hypothesis, expression pattern, subcellular localization, and transport activities toward metals (including Cd, Mn, Co, Fe, and Zn) of TpNRAMP3 were investigated.

## Materials and Methods

### RNA Sample Collection, Isolation, and cDNA Synthesis

For expression pattern of *TpNRAMP3* grown in normal wheat growth season, 5 (root, basal stem, leaf sheath, leaf blade, and young leaf), 11 (root, basal stem, low leaf sheath, low leaf blade, node III, inter node II, node II, node I, flag leaf sheath, flag leaf blade, and peduncle), and 7 tissues (root, node I, flag leaf sheath, flag leaf blade, inter node I, lemma, and immature grain) were individually collected at jointing, booting, and grain filling stages with three biological replicates.

For response to metal stresses grown in Hoagland nutrient solution in a growth chamber at 25°C with 16/8 h light/dark, seedlings of DPW at the three-leaf stage were treated with 8 mM MgCl_2_ (Mg), 8 mM ZnSO_4_ (Zn), 8 mM FeCl_3_ (Fe), 8 mM CuCl_2_ (Cu), 40 μM CdSO_4_ (Cd), 40 μM PbCl_2_ (Pb), or 40 μM NiCl_2_ (Ni) for 24 h. Roots and leaves were separately collected (three biological replicates).

All collected samples were snap-frozen in liquid nitrogen and stored at -80°C. The Total RNA Kit II (Omega, United States) was used to isolate total RNA of each sample. Meanwhile, cDNA was synthesized from 2 μg total RNA using the M-MLV First Strand cDNA Synthesis Kit (Omega, United States).

### TpNRAMP3 Cloning

PCR primers (F: 5′-TCATTGGGAGAGAGTGAGCAT-3′; R: 5′-ACATATCTAGTTT CCTCGCTGC-3′) for the full-length cDNA of *TpNRAMP3* were designed according to DPW transcript (Wang et al., 2016). PCR volume included 2.5 μl 10× buffer, 2.0 μl MgCl_2_ (25 mM), 2.0 μl dNTP mixture (2.5 Mm each), 2.0 μl forward primer (4 pM), 2.0 μl reverse primer (4 pM), 3.0 μl cDNA, 0.5 μl ExTaq, and added sterilized distilled water up to 25 μl. PCR conditions were 95°C for 3 min, 30 cycles (95°C for 30 s, 60°C for 30 s, 72°C for 3 min), 72°C for 10 min, and 4°C hold. The amplified fragment was cloned into the pMD19-T vector for sequencing, and the results were analyzed with Invitrogen Vector NTI 11.5.1 (Invitrogen, United States).

### Bioinformatics and Phylogenetic Analysis

Amino acid sequence, gene structure and chromosome localization, putative subcellular localization, transmembrane (TM) domains and phylogenetic analysis of TpNRAMP3 were performed as described by Peng et al. (2018). Protein structures of TpNRAMP3 and OsNRAMP3 were predicted using I-TASSER^1^, which used the crystal structure of *Eco*NRAMP (from *Eremococcus coleocola*) as a model (Ehrnstorfer et al., 2017). The CIS regulatory elements of the promoters of *TpNRAMP3* and *OsNRAMP3* were predicted using PlantCARE^2^.

### Expression Analysis of *TpNRAMP3*

Quantitative real-time PCR with *TpNRAMP3*-specific primers (forward: 5′-ACTCTGATGCTCCTGTTCCT-3′; reverse: 5′-GCCTCGCACAACTTCTGAA-3′) was performed as described by Wang et al. (2015) with nine technological replicates of each sample. The *actin* gene (Wang et al., 2015) was used as a reference gene to normalize relative expression level of *TpNRAMP3* which was calculated by CFX Manager 3.1 (Bio-Rad, United States) using the 2^ΔΔ^*^C^*^t^ method.

### Expression in Yeast

The open reading frame of *TpNRAMP3* was inserted into the pYES2 vector at the *Bam*HI and *Eco*RI sites. The recombinant plasmid and the empty vector were transformed into different yeast strains including wild type (WT) BY4743, Cd-sensitive mutant Δ*ycf1*, Co-sensitive mutant *YK40* and Zn-sensitive mutant Δ*zrc1* as described by Peng et al. (2018). The positively transformed yeasts were selected on SD solid medium (6.7 g/L yeast nitrogen base without amino acids, 1.92 g/L yeast synthetic drop-out medium supplements, 20 g/L bacteriological agar and 20 g/L glucose) with ampicillin, and were confirmed by PCR using *TpNRAMP3*-specific primers.

Positively transformed yeast cells were grown in liquid SD medium at 30°C for 16 h. Each cell suspension was diluted to OD_600_ = 0.8 and then subjected to four sequential dilutions (1:10, 1:100, 1:1000, and 1:10000). Five microliters of each diluted cell suspension was spotted on plates of SD medium containing CdCl_2_ (0 or 80 μM), CoCl_2_ (0 or 200 μM), or ZnSO_4_ (0 or 4 mM), with 2% galactose. All plates were incubated at 30°C for 3 days to compare their sensitivity to Cd, Co, and Zn with three replicates of each metal stress.

To confirm metal tolerance, yeast growth (the OD_600_ value) was quantified at different times. 50 μl of positive yeast cells with OD_600_ = 0.8 were cultured in 10 mL SD liquid medium with 2% galactose and CdCl_2_ (0, 20, or 40 μM) or CoCl_2_ (0, 200, or 400 μM). For Cd sensitivity, OD_600_ values were observed at 0, 6, and 24 h; for Co sensitivity, OD_600_ values were observed at 0, 12, and 36 h. All values were measured using a microplate spectrophotometer (Fisher Scientific, United States) with three replicates.

Meanwhile, Cd and Co concentration of yeast was measured to understand whether TpNRAMP3 transports Cd or Co in yeast. Positively transformed cells were grown in SD liquid medium with CdCl_2_ (20 μM) for 48 h or CoCl_2_ (50 μM) for 60 h. Metal concentration of each sample was determined by inductively coupled plasma-mass spectrometry (ICP-MS, Fisher Scientific, United States) as described by Peng et al. (2018) with three biological replicates.

### Subcellular Localization of TpNRAMP3 in *Arabidopsis* Protoplasts

The open reading frame of *TpNRAMP3* was sub-cloned into the *Arabidopsis* protoplast expression vector HBT95-green fluorescence protein (GFP) at the *Bam*HI and *Spe*I sites under the 35S promoter. As described by Yoo et al. (2007), *Arabidopsis* mesophyll protoplast was prepared and transformed. Meanwhile, the plasma membrane marker, RFP-SCAMP1 (Cai et al., 2011), was used to confirm the subcellular localization. A confocal laser scanning microscope (Olympus, Japan) was used to detect GFP and RFP signals.

### Overexpression of *TpNRAMP3* in *Arabidopsis thaliana*

The open reading frame of *TpNRAMP3* was sub-cloned into the pCAMBIA1305.1 vector at the *Bam*HI and *Sal*I sites under the CaMV35s promoter. The recombined vector and the empty vector were individually introduced into *Agrobacterium*, and then transformed into WT *Arabidopsis thaliana* plants using floral infiltration (three time-independent transformations). The positive and homozygous lines were selected using hygromycin selection and PCR with *TpNRAMP3*-specific primers. The overexpression level was analyzed as described as the section of “Expression analysis of *TpNRAMP3*.”

To test metal tolerance of TpNRAMP3 in transformed seedlings, the WT, an empty vector line and two independently homozygous lines (each line contained three plant lines selected from an independent transformation; totally six plant lines) were seeded on 1/2 MS solid plates containing 25 μM CdCl_2_, 80 μM CoCl_2_, or 500 μM MnCl_2_. After 4°C treatment for 24 h, the plates were placed in a light incubator with 120 μE m^-2^ s^-1^ illumination intensity, a 16/8 h light/dark period, 22°C temperature, and 50% humidity. On the 10th day, the root lengths of the treated plants were determined with three biological replicates.

To investigate metal transport properties of TpNRAMP3 in plant, the WT, an empty vector line and two independent homozygous lines were germinated on 1/2 MS medium. Seedlings were transplanted into soil-filling plot at the four-leaf stage (12 plots of each WT and empty line; 36 plots of each independent homozygous line). After 3 weeks, 100 mg CdCl_2_ or CoCl_2_ was added to 2.5 kg soil (four plots of each metal stress of each line). At the mature stage, roots and aerial parts were individually collected and dried at 60°C for 48 h with three biological replicates. Dry weight of each plant was calculated using the formula [(root dry weight + aerial part dry weight)/collected plant number]. All dried samples were digested in 80% nitric acid at 220–280°C, and diluted in deionized water. Metal concentration of each sample was determined using ICP-MS (Fisher Scientific, United States). Translocation factor (TF), representing metal translocation from roots to shoots, was calculated by the shoot-to-root concentration ratio.

### Data Analysis

Differences of samples or treatments were analyzed using SPSS 20.0 with Tukey’s test at *P* ≤ 0.05. All figures were drawn using Sigmaplot 12.0.

## Results

### Cloning and Phylogenetic Analysis of *TpNRAMP3*

The full-length cDNA of *TpNRAMP3* (KX165384) including a 19-bp 5′-UTR, 1644-bp open reading frame, and 36-bp 3′-UTR was successfully amplified. It encoded 548 amino acids (ANT73691), which had 90.0% identity to OsNRAMP3, 72.1% identity to AtNRAMP1 and 71.2% identity to AtNRAMP6 (**Supplementary Figure S1**). Blasting the cDNA of *TpNRAMP3* against the wheat genome (The International Wheat Genome Sequencing Consortium, 2014) revealed that *TpNRAMP3* was localized on the chromosome 7BL (gene: TRIAE_CS42_7BL_TGACv1_576780_AA1854780). It comprised 12 introns and 13 exons. Compared with structure of OsNRAMP3, TpNRAMP3 had a longer α-helix in its C-terminus (**Supplementary Figure S2**), which implied that its function might differ from that of OsNRAMP3.

### *TpNRAMP3* Expression

Before investigation of the expression pattern of *TpNRAMP3*, we compared the promoters of *TpNRAMP3* and *OsNRAMP3* to search for distinct CIS regulatory elements. Ten *TpNRAMP3*-specific and five *OsNRAMP3*-specific CIS regulatory elements were individually predicted from the promoters of *TpNRAMP3* and *OsNRAMP3* (**Supplementary Table 1**). These distinct CIS regulatory elements between the promoters of *TpNRAMP3* and *OsNRAMP*3 implied that the expression pattern of *TpNRAMP3* might differ from *OsNRAMP3*.

**FIGURE 1:**
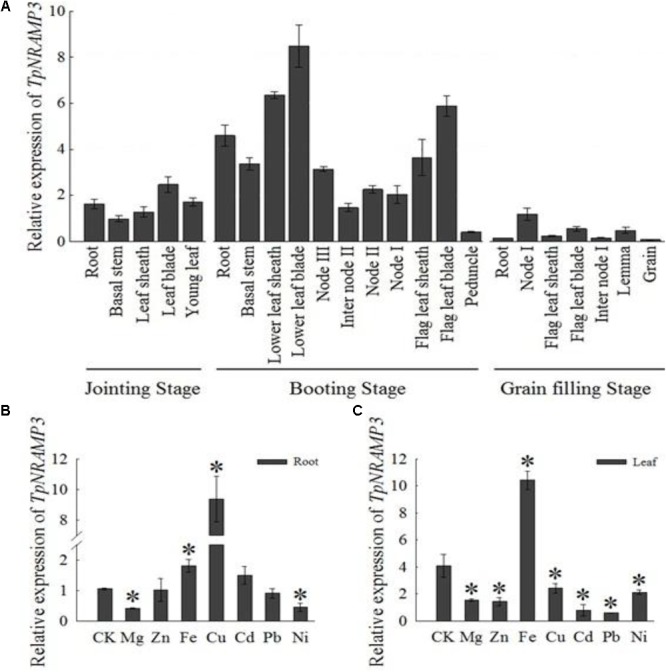
Expression pattern of *TpNRAMP3*. **(A)** At different growth stages, the relative expression of *TpNRAMP3* in various tissues that were collected from DPW grown in a field at natural wheat growth season **(B,C)**: the relative expression of *TpNRAMP3* in roots **(B)** and shoots **(C)** that treated with Mg, Zn, Fe, Cu, Cd, Pb, or Ni for 24 h. Asterisk indicated significant difference from control at *P* < 0.05 by Tukey’s test with three independently biological replicates. Jointing stage: the basal stem emerges above the soil line; booting stage: the developing head with the sheath of the flag leaf becomes visibly enlarged; internode I: the first internode below the spike; internode II: the second internode below the spike; node I: the node with the flag leaf; node II: the first node below the node I; node III: the second node below the node I. CK was treated without supplementation with metals.

The expression pattern of *TpNRAMP3* was investigated in different tissues at three growth stages of wheat grown in a field. At the jointing stage, *TpNRAMP3* showed the highest expression in leaf blades, followed by roots and new leaves, and the lowest expression in basal stems and leaf sheaths (**Figure 1A**). At the booting stage, the expression of *TpNRAMP3* was highest in old leaf blades, followed by old leaf sheaths and flag leaf blades, then in roots and flag leaf sheaths, and lowest in immature spikes (**Figure 1A**). At the grain filling stage, *TpNRAMP3* expression was highest in the first nodes (node I) and lowest in roots and grains (**Figure 1A**).

We also investigated the responses of *TpNRAMP3* to supplementation with metals including Mg, Zn, Fe, Cu, Cd, Pb, and Ni. In the roots, *TpNRAMP3* expression was significantly down-regulated by Mg and Ni, and up-regulated by Fe and Cu, but not affected by Zn, Cd, or Pb (**Figure 1B**). However, *TpNRAMP3* expression was affected by all metal stresses in the shoots (**Figure 1C**).

### Subcellular Localization of TpNRAMP3

**FIGURE 2:**
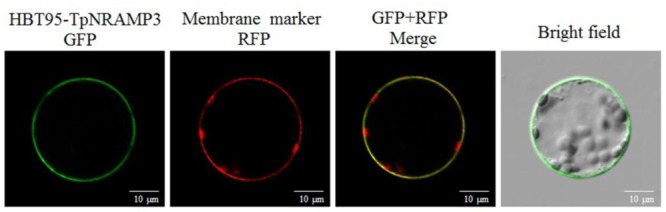
Subcellular localization of TpNRAMP3. A plasma membrane marker (RFP-SCAMP1) was used in this study. TpNRAMP3 was localized at the plasma membrane.

TpNRAMP3 was predicted to be a plasma membrane protein with 11 TM domains (**Supplementary Figure S3**). To confirm the subcellular localization of TpNRAMP3, a TpNRAMP3-GFP fusion protein and a plasma membrane marker were transiently co-transformed into *Arabidopsis* leaf protoplasts. Green fluorescence from the empty vector (HBT95) was localized in the cytosol, nucleus, and plasma membrane (Wang et al., 2018). However, green fluorescence from the fusion protein (HBT95-TpNRAMP3-GFP) was mostly merged with the red fluorescence of the membrane marker, which indicated that TpNRAMP3 was localized at the plasma membrane (**Figure 2**).

### Metal Transport in Yeast

**FIGURE 3:**
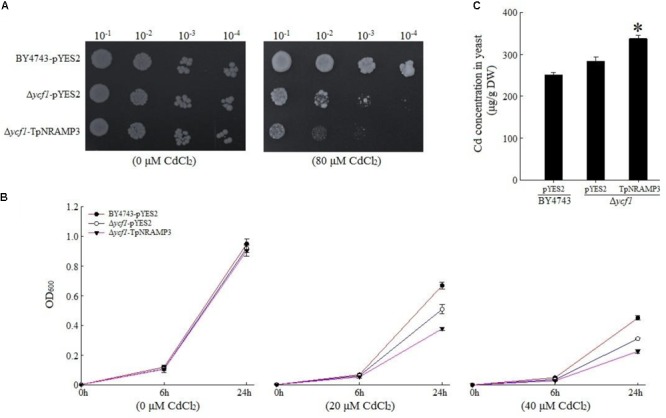
Sensitivity to Cd and Cd concentration in yeast. **(A)** Sensitivity to Cd when grown on plate SD medium with 80 μM CdCl_2_ **(B)** sensitivity to Cd when grown in liquid SD medium with 20 and 40 μM CdCl_2_; **(C)** Cd concentration in yeast when grown in SD medium with 20 μM CdCl_2_ for 48 h. Asterisk indicated significant difference when compared with WT at *P* < 0.05; value was mean ± standard deviation (three biological replicates).

**FIGURE 4:**
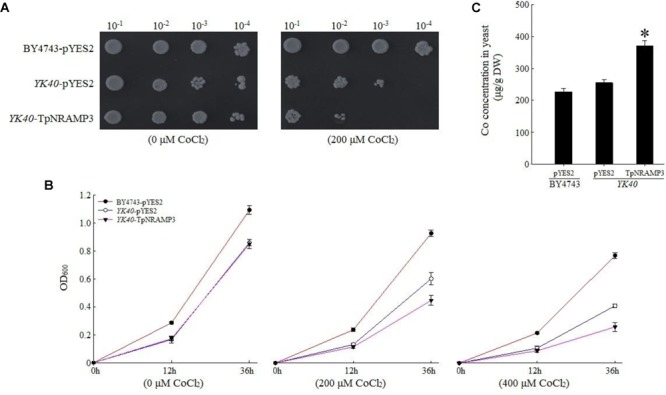
Sensitivity to Co and the Co concentration in yeast. **(A)** Sensitivity to Co when grown on plate SD medium with 200 μM CoCl_2_; **(B)** sensitivity to Co when grown in liquid SD medium with 200 and 400 μM CdCl_2_; **(C)** Co concentration in yeast when grown in SD medium with 50 μM CoCl_2_ for 60 h. Asterisk indicated significant difference when compared with WT at *P* < 0.05; value was mean ± standard deviation (three biological replicates).

To understand TpNRAMP3’s function, we firstly expressed *TpNRAMP3* in Cd-, Co-, and Zn-sensitive mutant yeasts for metal transport assays. In the presence of galactose, no growth difference was observed among the transformed yeasts under 0 μM Cd stress (**Figures 3A,B**). The expression of *TpNRAMP3* significantly inhibited the growth of *Δycf1* strains compared with *Δycf1* strains transformed with pYES2 under Cd stress (**Figure 3A**). The growth inhibition induced by *TpNRAMP3* was confirmed by the growth curves under 20 and 40 μM CdCl_2_ stresses (at 6 and 24 h) (**Figure 3B**). Furthermore, the Cd concentrations in the yeast cells were investigated. Δ*ycf1* cells expressing *TpNRAMP3* had significantly higher Cd concentration than cells transformed with pYES2 (**Figure 3C**). Similar results were observed under Co stress (**Figure 4**). The expression of *TpNRAMP3* significantly inhibited the growth of *YK40* strains whether they were grown on SD plates with 200 μM Co (**Figure 4A**) or SD liquid medium with 200 or 400 μM Co (**Figure 4B**). *TpNRAMP3* expression also significantly increased Co concentration under 50 μM Co stress compared with yeast cells transformed with pYES2 (**Figure 4C**). These results indicated that TpNRAMP3 is a Cd and Co transporter. However, Zn did not affect yeast growth regardless of whether the cells were expressing *TpNRAMP3* or transformed with pYES2 (**Supplementary Figure S4**).

### Functional Analysis of TpNRAMP3 in *Arabidopsis*

**FIGURE 5:**
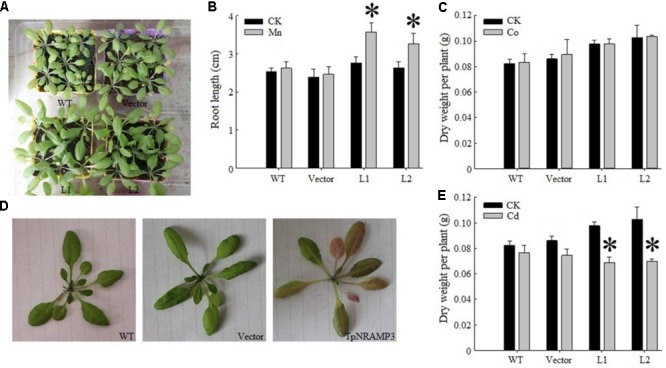
Phenotypes of *TpNRAMP3*-expressing *Arabidopsis* treated with different metals. **(A)** Expression of *TpNRAMP3* slightly improved the plant growth grown in soil for 4 weeks; **(B)** expression of *TpNRAMP3* significantly increased the length of seedling root on 1/2 MS medium with 500 μM MnCl_2_ for 10 days; **(C)** expression of *TpNRAMP3* did not affect the dry weight of adult plant when grown in soil with 40 mg/kg Co stress; **(D,E)** 40 mg/kg Cd caused the Cd toxicity in the leaves **(D)** and reduced the dry weight of adult plant **(E)** of *TpNRAMP3*-expressing lines. Asterisk indicated significant difference when compared with their individual control (CK) at *P* < 0.05; value was mean ± standard deviation (three biological replicates).

To determine whether TpNRAMP3 transports Cd and Co, or other metals such as Fe and Mn in plants, we expressed *TpNRAMP3* in *Arabidopsis* under the 35S promoter. Two independent homozygous overexpression lines were developed (**Supplementary Figure S5A**). Under normal growth conditions, expression of *TpNRAMP3* increased plant growth compared with the WT and empty vector line (**Figure 5A**). Under 500 μM MnCl_2_ stress, expression of *TpNRAMP3* dramatically increased seedling root lengths (**Figure 5B**). Under 25 μM CdCl_2_ and 80 μM CoCl_2_ stresses, expression of *TpNRAMP3* did not affect root lengths (**Supplementary Figures S5B,C**). However, whether plants were under 40 mg/Kg CoCl_2_ stress or not, expression of *TpNRAMP3* slightly increased dry weight per plant (**Figure 5C**). Although 40 mg/kg CdCl_2_ did not cause Cd-toxicity in the WT and empty vector line, it did cause Cd-toxicity (red speckles on the leaves, **Figure 5D**) and reduce the dry weight per plant in *TpNRAMP3*-expressing lines compared with their individual controls (CK) (**Figure 5E**).

**FIGURE 6:**
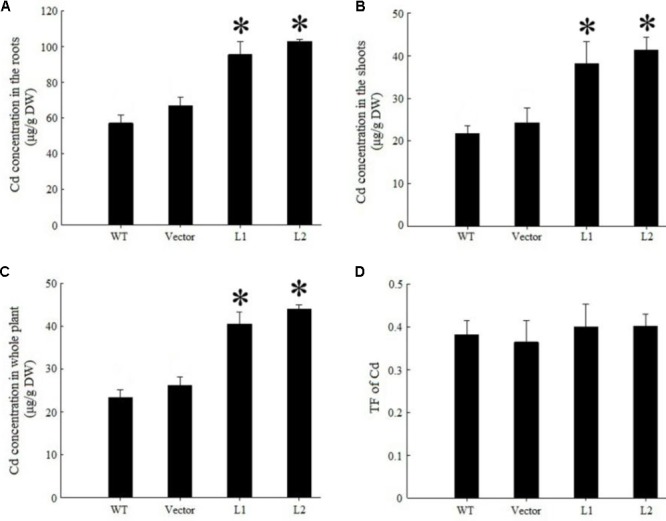
Cd concentration and translocation factor of *TpNRAMP3*-expressing *Arabidopsis*. **(A–C)** Cd concentration in roots, shoots, and whole plant, respectively; **(D)** Cd translocation factor (TF) represented Cd translocation from roots to shoots. Asterisk indicated significant difference when compared with WT at *P* < 0.05; value was mean ± standard deviation (three biological replicates).

**FIGURE 7:**
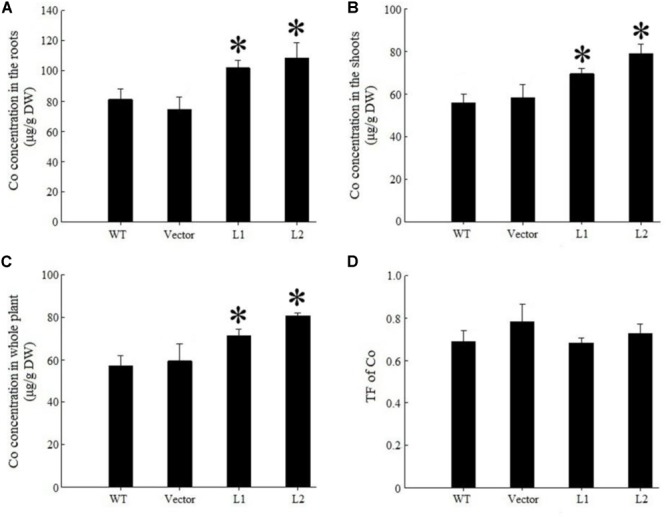
Co concentration and TF of *TpNRAMP3*-expressing *Arabidopsis*. **(A–C)** Co concentration in roots, shoots, and whole plant, respectively; **(D)** Co TF. Asterisk indicated significant difference when compared with WT at *P* < 0.05; value was mean ± standard deviation (three biological replicates).

**FIGURE 8:**
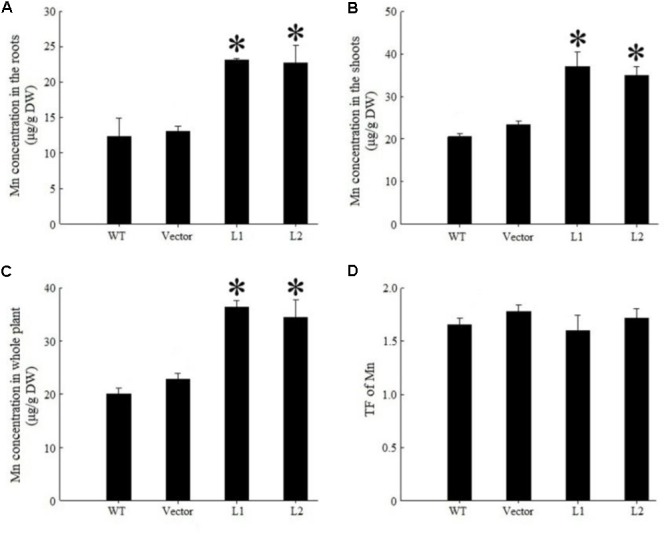
Mn concentration and TF of *TpNRAMP3*-expressing *Arabidopsis*. **(A–C)** Mn concentration in roots, shoots, and whole plant, respectively; **(D)** Mn TF. Asterisk indicated significant difference when compared with WT at *P* < 0.05; value was mean ± standard deviation (three biological replicates).

Under 40 mg/kg CdCl_2_ stress, expression of *TpNRAMP3* significantly increased Cd concentration in roots (**Figure 6A**), shoots (**Figure 6B**) and whole plant (**Figure 6C**) compared with the WT and empty vector line. However, it did not affect Cd translocation from roots to shoots (**Figure 6D**). Under 40 mg/kg CoCl_2_ stress, expression of *TpNRAMP3* significantly increased Co concentration in roots (**Figure 7A**), shoots (**Figure 7B**), and whole plant (**Figure 7C**), but did not affect Co translocation from roots to shoots (**Figure 7D**). Under normal growth conditions, expression of *TpNRAMP3* also dramatically increased Mn concentration in roots (**Figure 8A**), shoots (**Figure 8B**), and whole plant (**Figure 8C**), but did not affect Mn translocation from roots to shoots (**Figure 8D**). However, expression of *TpNRAMP3* did not affect Fe or Zn concentration in roots (**Supplementary Figures S6A,C**) or shoots (**Supplementary Figures S6B,D**). These results indicated that TpNRAMP3 is a metal transporter for Cd, Co, and Mn accumulation, but not for Fe or Zn accumulation.

## Discussion

### TpNRAMP3 Is a Metal Transporter for Co, Mn, and Cd Accumulation, but Does Not Transport Fe or Zn

In the present study, we found that TpNRAMP3 is a Co, Mn, and Cd transporter, and is potentially responsible for Co, Mn, and Cd accumulation in wheat. This conclusion is based on the following evidence: (1) although *TpNRAMP3* was mainly expressed in leaf blade at the jointing stage, and older leaf blade and sheath, and flag leaf blade at the booting stage, it was also highly expressed in roots at these stages (**Figure 1A**); (2) TpNRAMP3 was localized at the plasma membrane (**Figure 2**); (3) expression of *TpNRAMP3* increased sensitivity to Co and Cd, and increased Co and Cd accumulation in yeast (**Figures 3, 4**); (4) expression of *TpNRAMP3* in *Arabidopsis* increased Cd (**Figure 6**), Co (**Figure 7**) and Mn (**Figure 8**) concentrations in roots, shoots and whole plant, and caused Cd-toxicity in leaves (**Figure 5D**) and reduced dry weight (**Figure 5E**). However, expression of *TpNRAMP3* did not alter Fe or Zn concentration in *Arabidopsis* (**Supplementary Figure S6**) or Zn sensitivity in yeast (**Supplementary Figure S4**).

Mn is an essential micronutrient element for plant growth and development (Socha and Guerinot, 2014). It is involved in many processes, such as photosynthesis, respiration, protein synthesis, and hormone activation (Shao et al., 2017). However, the uptake, translocation and sequestration of Mn may share transporters with Fe. In rice, yellow stripe 1-like 2 (OsYSL2) is implicated in Fe and Mn transport (Koike et al., 2004). Expression of *OsNRAMP6* showed Fe and Mn transport activity in yeast (Gao et al., 2018). Additionally, knockout of *OsNRAMP5*, which localizes to the root plasma membrane, reduced Fe and Mn uptake (Ishimaru et al., 2012; Sasaki et al., 2012). In *Arabidopsis*, AtNRAMP1 localizes at the plasma membrane and is responsible for Mn uptake (Cailliatte et al., 2010) and Fe homoeostasis (Curie et al., 2000). In this study, expression of *TpNRAMP3* in *Arabidopsis* dramatically increased Mn accumulation in roots (**Figure 8A**), shoots (**Figure 8B**), and whole plant (**Figure 8C**), reasonably explaining why expression of *TpNRAMP3* promoted the growth of plants in normal soils (**Figure 5A**) and on MS medium with 500 μM MnCl_2_ (**Figure 5B**). However, expression of *TpNRAMP3* in *Arabidopsis* did not alter Fe concentration in roots or shoots (**Supplementary Figures S6A,B**). Mn accumulation therefore has its own transporter in wheat, and does not share this transporter with Fe.

Co is a non-essential nutrient for plants, which is taken up from soil by IRT1 in *Arabidopsis* (Korshunova et al., 1999; Barberon et al., 2014). The absorbed Co is sequestrated in root vacuoles by ferroportins IRON REGULATED2 (IREG2/FPN2) (Morrissey et al., 2009) and AtHMA3 (Morel et al., 2009). Additionally, it is loaded into the xylem by IREG1/FPN1 and transported to the shoot (Morrissey et al., 2009; Lange et al., 2017). IRT1, IREG1/FPN1, and IREG2/FPN2 also function as Fe transporters; therefore, the uptake and translocation of Co is also Fe-regulated (Morrissey et al., 2009). In the present study, although TpNRAMP3 increased Co accumulation in yeast (**Figure 4C**) and *Arabidopsis* (**Figures 7A–C**), it did not alter Co translocation (**Figure 7D**), and did not change Fe or Zn concentration in *Arabidopsis* (**Supplementary Figure S6**). Thus, its function is different from those of IREG2/FPN2 (Morrissey et al., 2009), AtHMA3 (Morel et al., 2009), IRT1 (Korshunova et al., 1999; Barberon et al., 2014), and IREG1/FPN1 (Morrissey et al., 2009; Lange et al., 2017).

Cd is also a toxic heavy metal that affects plant growth and development. Theoretically, no specific transporter is responsible for Cd uptake and translocation (Sasaki et al., 2012). Because of the similar physical and chemical characteristics between Cd and essential metals including Zn and Fe (Chesworth, 1991), they may share transporters (Sasaki et al., 2012). For example, OsNRAMP1 and OsNRAMP5 transport Fe and Cd in plants and/or yeast (Takahashi et al., 2011; Ishimaru et al., 2012; Sasaki et al., 2012; Takahashi et al., 2014). However, some transporters that transport Cd but not Fe have been discovered, such as AtNRAMP6 (Peris-Peris et al., 2017) and HvNRAMP5 (Wu et al., 2016). In this study, we found that TpNRAMP3 was a Cd transporter responsible for Cd accumulation (**Figures 3C, 6**), but did not accumulate Fe or Zn (**Supplementary Figure S6**). Its function is different from that of HvNRAMP5, because *HvNRAMP5* is mainly expressed in the roots (Wu et al., 2016).

Wheat flour is the main contributor to the average daily dietary intake of Cd (Greger and Löfstedt, 2004). Therefore, limiting Cd accumulation in wheat grains is necessary to protect human health. Cd accumulation in wheat grains is controlled by the root uptake of Cd, the translocation of Cd from the roots to shoots, and the distribution of Cd in the leaves and grain coat (Greger and Löfstedt, 2004). Thus, reducing Cd uptake is the first and most important step to reduce Cd accumulation in wheat grains. Since TpNRAMP3 potentially increases Cd and Mn accumulation in wheat, knockout or knockdown of *TpNRAMP3* will reduce Cd transport from the soil to wheat grains in the future.

### *TpNRAMP3* and *OsNRAMP3* Have Different Expression Patterns and Metal Transport Functions

There was 90% identity of amino acid sequence between TpNRAMP3 and OsNRAMP3, which implied that functional divergence between TpNRAMP3 and OsNRAMP3. Previous studies indicated that *OsNRAMP3* was mainly expressed in nodes, culms, and basal stems, with very low expression in roots and leaf blades and sheaths (Yamaji et al., 2013; Yang et al., 2013). Additionally, the expression of *OsNRAMP3* was unaffected by Fe, Zn, Cu, or Mn deficiency (Yamaji et al., 2013; Yang et al., 2013). In this study, *TpNRAMP3* was mainly expressed in leaf blades, leaf sheaths, and roots, but had very low expression in nodes and internodes at the jointing and booting stages (**Figure 1A**). Interestingly, *TpNRAMP3* was mainly expressed in the first node at the grain filling stage (**Figure 1A**). Additionally, the expression of *TpNRAMP3* was regulated by many metals including Fe, Zn, Cu, Pb, Ni, Cd, and Mg (**Figure 1B**). Thus, the expression patterns of *OsNRAMP3* and *TpNRAMP3* differ, which might be results of distinct sequences (**Supplementary Figure S3**), CIS regulatory elements of the promoters (**Supplementary Table 1**), and/or species.

In rice, OsNRAMP3 functions as a switch in response to environmental Mn changes (Yamaji et al., 2013). At low Mn concentrations, OsNRAMP3 transports Mn to young leaves and panicles; however, at high Mn concentrations, Mn transport to old leaves is caused by OsNRAMP3 degradation (Yamaji et al., 2013). Thus, OsNRAMP3 is a Mn transporter responsible for Mn distribution and contributes to the remobilization of Mn from old to young leaves (Yamaji et al., 2013; Yang et al., 2013). Although the amino acid sequence of TpNRAMP3 has high identity (90%) to that of OsNRAMP3 (**Supplementary Figure S1**), our results suggest their metal transport functions are different. This conclusion is supported by the following: (1) TpNRAMP3 functions as a Cd and Co transporter in yeast (**Figures 3, 4**) and *Arabidopsis* (**Figures 6, 7**), but OsNRAMP3 is not a Cd transporter (Yamaji et al., 2013); and (2) *TpNRAMP3* expression increased Mn concentrations in roots (**Figure 8A**), shoots (**Figure 8B**), and whole plant (**Figure 8C**), while knockout of *OsNRAMP3* did not affect Mn concentration in roots or shoots (Yamaji et al., 2013; Yang et al., 2013). These differences are probably caused by a few residues in the primary sequences of TpNRAMP3 and OsNRAMP3 (**Supplementary Figure S3**), because residue differences change the putative protein structures (**Supplementary Figure S2**) and can alter metal transport substrates, such as in AtNRAMP4 (Pottier et al., 2015) and NtNRAMP5 (Tang et al., 2017).

## Conclusion

*TpNRAMP3* is localized on the chromosome 7BL. It was highly expressed in leaf blades and roots at the jointing and the booting stages, and in the first nodes at the grain filling stage. TpNRAMP3 was localized at the plasma membrane. The expression of *TpNRAMP3* was significantly down-regulated by Mg and Ni, and up-regulated by Fe and Cu in seedling roots, but not affected by Zn, Cd, or Pb. In yeast, TpNRAMP3 functions as a metal transporter for Cd and Co accumulation, but not Zn. Expression of *TpNRAMP3* in *Arabidopsis* showed that TpNRAMP3 is a metal transporter for Cd, Co, and Mn accumulation. Thus, the function of TpNRAMP3 is different from that of OsNRAMP3, which functions as a Mn switch (Yamaji et al., 2013; Yang et al., 2013), OsNRAMP1, which is involved in Cd (but not Mn) uptake and translocation from roots to shoots (Takahashi et al., 2011; Tiwari et al., 2014), and OsNRAMP5, which functions as a Cd, Mn, and Fe transporter (Ishimaru et al., 2012; Sasaki et al., 2012; Takahashi et al., 2014).

## Author Contributions

FP, CW, and YC conducted all the experiments. YW, FP, YZ, and JZ designed the manuscript. FP, CW, YW, HK, XF, and HZ analyzed the data. FP, CW, YW, YZ, YC, and LS drafted and revised the manuscript.

## Conflict of Interest Statement

The authors declare that the research was conducted in the absence of any commercial or financial relationships that could be construed as a potential conflict of interest.

## Footnotes

1 https://zhanglab.ccmb.med.umich.edu/I-TASSER/

2 http://bioinformatics.psb.ugent.be/webtools/plantcare/html/
