# Supplementary material for: Cloning and Characterization of TpNRAMP3, a Metal Transporter From Polish Wheat (Triticum polonicum L.)
Source: Front Plant Sci. 2018 Sep 20;9:1354. doi: 10.3389/fpls.2018.01354 (PMC6158329; doi:10.3389/fpls.2018.01354)
Supplement: Supplementary file 1 [file Table_1.pdf]

Supplementary Table 1 The CIS regulatory elements between TpNRAMP3 and OsNRAMP3.

Gene	Cis-acting element	Function
TpNRAMP3	3-AF1 binding site	Light responsive element
	5’ UTR Py-rich stretch	Element conferring high transcription levels
	A-box	Cis-acting regulatory element
	ABRE	Cis-acting element involved in the abscisic acid responsiveness
	AE-box	Part of a module for light response
	CAAT-box	Common cis-acting element in promoter and enhancer regions
	CAT-box	Cis-acting regulatory element related to meristem expression
	CCGTCC-box	Cis-acting regulatory element related to meristem specific activation
	CGTCA-motif	Cis-acting regulatory element involved in the MeJA- responsiveness
	G-box	Cis-acting regulatory element involved in light responsiveness
	GAG-motif	Part of a light responsive element
	GARE-motif	Gibberellin-responsive element
	GCN4_motif	Cs-regulatory element involved in endosperm expression
	GT1-motif	Light responsive element
	I-box	Part of a light responsive element
	L-box	Part of a light responsive element
	Skn-1_motif	Cis-acting regulatory element required for endosperm expression
	Sp1	Light responsive element
	TATA-box	Core promoter element around -30 of transcription start

	TATC-box	Cis-acting element involved in gibberellin-responsiveness
	TC-rich repeats	Cis-acting element involved in defense and stress responsiveness
	TCA-element	Cis-acting element involved in salicylic acid responsiveness
	TCT-motif	Part of a light responsive element
	TGACG-motif	Cis-acting regulatory element involved in the MeJA-responsiveness
OsNRAMP3	ABRE	Cis-acting element involved in the abscisic acid responsiveness
	ACE	Cis-acting element involved in light responsiveness
	ATCT-motif	Part of a conserved DNA module involved in light responsiveness
	CAAT-box	Common cis-acting element in promoter and enhancer regions
	CAT-box	Cis-acting regulatory element related to meristem expression
	CGTCA-motif	Cis-acting regulatory element involved in the MeJA- responsiveness
	G-box	Cis-acting regulatory element involved in light responsiveness
	GAG-motif	Part of a light responsive element
	GARE-motif	Gibberellin-responsive element
	GCN4_motif	Cis-regulatory element involved in endosperm expression
	I-box	Part of a light responsive element
	MBS	MYB binding site involved in drought-inducibility
	Skn-1_motif	Cis-acting regulatory element required for endosperm expression
	Sp1	Light responsive element
	TATA-box	Core promoter element around -30 of transcription start
	TCT-motif	Part of a light responsive element
	TGACG-motif	Cis-acting regulatory element involved in the MeJA-responsiveness

	Circadian	Cis-acting regulatory element involved in circadian control
	rbcS-CMA7a	Part of a light responsive element

Note: yellow marked the *TpNRAMP3*-specific CIS regulatory elements; green marked the *OsNRAMP3*-specific CIS regulatory elements.

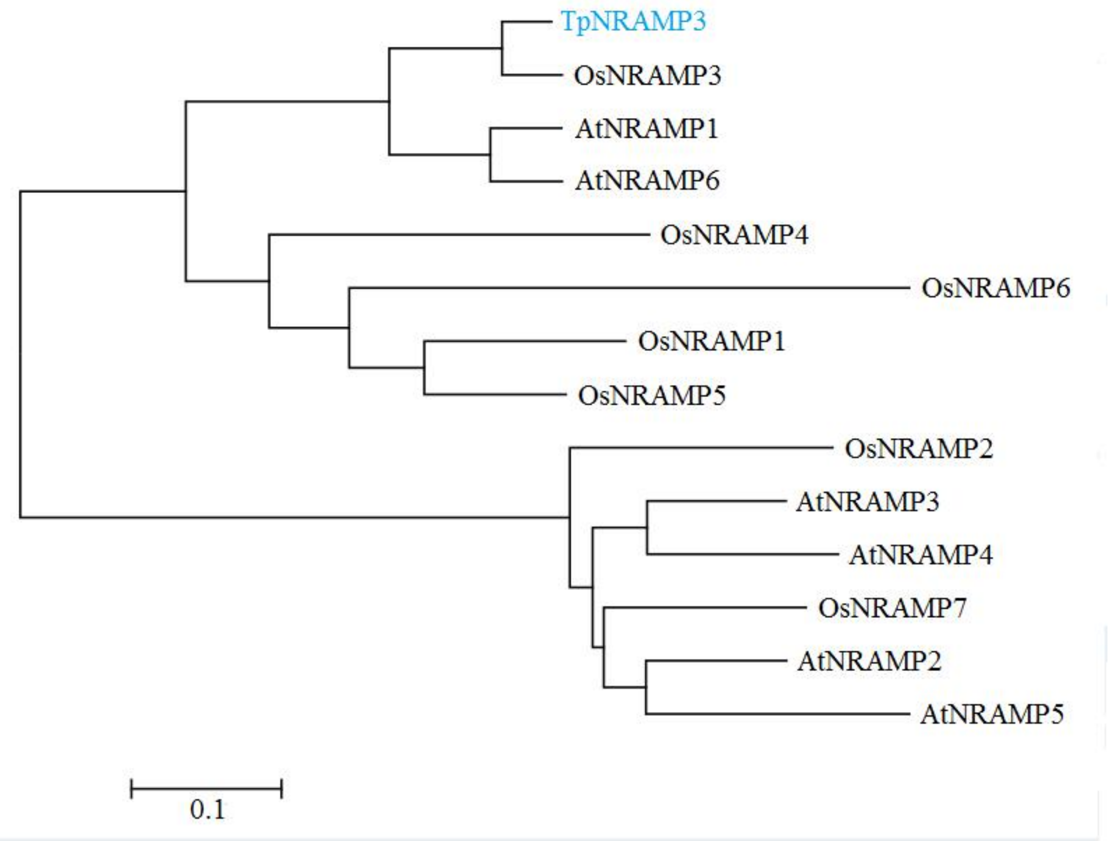

FIg S1. TpNRAMP3 is closely grouped with OsNRAMP3.

TpNRAMP3 (KX165384), 7 OsNRAMP proteins and 6 AtNRAMP proteins, including OsNRAMP1 (AK121534), OsNRAMP2 (L81152), OsNRAMP3 (AK070574), OsNRAMP4 (LOC_Os02g03900), OsNRAMP5 (AK070788), OsNRAMP6 (XM_015762723), OsNRAMP7 (LOC_Os12G39180), AtNRAMP1 (AF165125), AtNRAMP2 (AF141204), AtNRAMP3 (AF202539), AtNRAMP4 (AF202540), AtNRAMP5 (NM_117995.2), AtNRAMP6 (NM_101464.4), were aligned using Vector NTI 11.5.1; the phylogenetic tree of the NRAMP proteins was constructed using MEGA5.

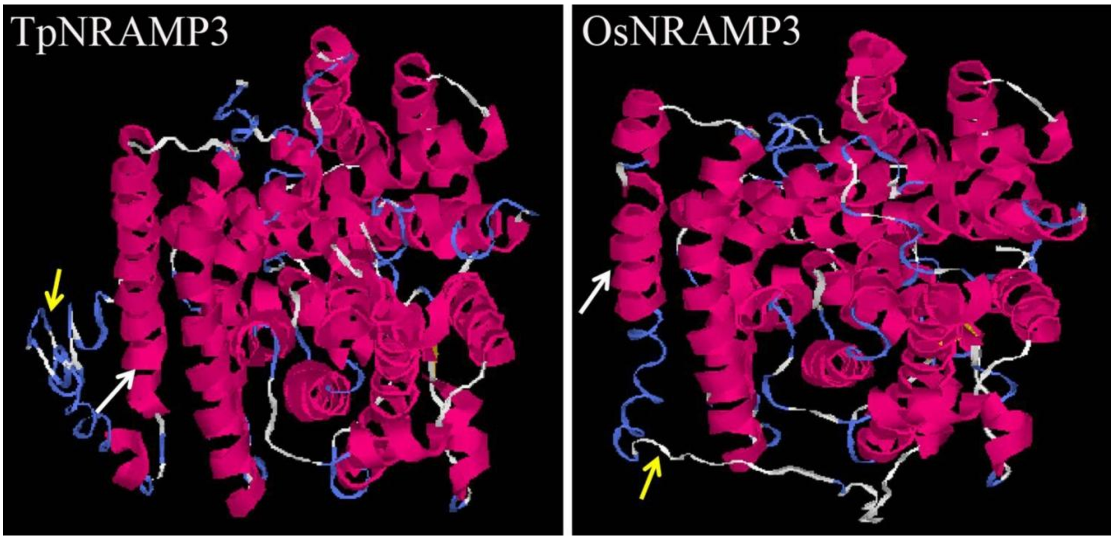

Fig S2. Protein structures of TpNRAMP3 and OsNRAMP3.

The protein structures of TpNRAMP3 and OsNRAMP3 were predicated using the software of I-TASSER. The white arrows represented the α-helix; the yellow arrows represented the randon coil.

```
OsNRAMP3  (1)   MSGPMQRSSQPQFISSVERNN-QSNGPGTPLIDSIDVDQIVIPEKNSWKN
TpNRAMP3  (1)   MSGPRQGSSQPQFMTSVGQNNNLSNGPGTPLIDSIDVDQIVIPEKNSWKN

OsNRAMP3  (50)  LFSYIGPGFLVSIAYIDPGNFETDLQAGAQYKYELLWIILIASCAALIIQ
TpNRAMP3  (51)  LFSYIGPGFLVSIAYIDPGNFETDLQAGAQYKYELLWIILIASCAALVIQ
                   TM1                                          TM2

OsNRAMP3  (100) SLAARLGVVTGKHLAEHCRAEYPKATNFILWILAELAVVACDIPEVIGTA
TpNRAMP3  (101) SLAASLGVVTGKHLAEHCRAEYPKVTNFILWILAELAVVACDIPEVIGTA
                                                  TM3

OsNRAMP3  (150) FALNMLFKIPVWCGVLITGLSTLMLLLLQQYGVRKLEFLIAILVSLIATC
TpNRAMP3  (151) FALNMLFKIPIWCGVLITGLSTLMLLFLQQYGVRKLEFLIAFLVFLIATC
                         TM4                                TM5

OsNRAMP3  (200) FLVELGYSKPNSSEVVRGLFVPELKGNGATGLAISLLGAMVMPHNLFLHS
TpNRAMP3  (201) FLVELGYSKPNSSEVVRGLFVPEIKGDGATGLAISLLGAMVMPHNLFLHS

OsNRAMP3  (250) ALVLSRKVPRSVHGIKEACRFYMIESAFALTIAFLINISIISVSGAVCGS
TpNRAMP3  (251) ALVLSRKVPRSVHGIKEACRFYMIESAFALTVAFLINISIISVSGAVCSA
                                                     TM6

OsNRAMP3  (300) DNLSPEDQMNCSDLDLNKASFLLKNVLGNWSSKLFAVALLASGQSSTITG
TpNRAMP3  (301) DNLNPEDRMNCNDLDLNKASFLLKNVLGNWSSKVFSIALLASGQSSTITG
                                                            TM7

OsNRAMP3  (350) TYAGQYVMQGFLDLRMTPWIRNLLTRSLAILPSLIVSIIGGSSAAGQLII
TpNRAMP3  (351) TYAGQYVMQGFLDLRMTPWLRNLLTRSLAIVPSLIVSLIGGSSAAGKLII
                                                  TM8

OsNRAMP3  (400) IASMILSFELPFALVPLLKFTSSRTKMGQHTNSKAISVITWGIGSFIVVI
TpNRAMP3  (401) IASMILSFELPFALVPLLKFTSSKTKMGPHTNSRFISVLTWAIGSFIMVI
                      TM9                                       TM10

OsNRAMP3  (450) NTYFLITSFVKLLLHNGLSTVSQVFSGIFGFLGMLIYMAAILYLVFRKNR
TpNRAMP3  (451) NIYFLITSFVRLLLHSGLSTVSQVFSGIFGFLGMLIYIAAILYLVFRKNR
                                                  TM11

OsNRAMP3  (500) KATLPLLEGDSTVRIVGRDTATEGEGSLGHLPREDISSMQLPQQRTASDL
TpNRAMP3  (501) KCTLPLLECDAKLGDAG---HTEGEGSLGHLPREDISSMQLPHQRPASDL

OsNRAMP3  (550) D
TpNRAMP3  (548) D
```

Fig S3. Alignments between TpNRAMP5 and OsNRAMP5.

Black lines marked the transmembrane domains predicted by SOSUI. The shaded residues represented their consensus positions were 100%, but their identity positions were 0%.

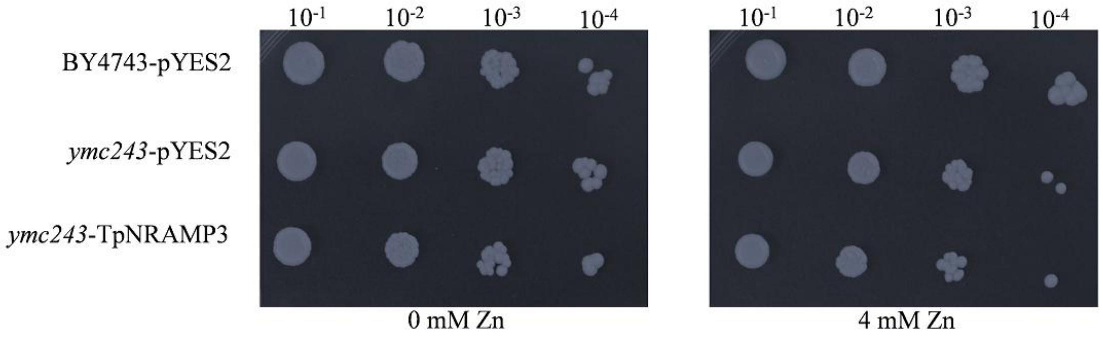

Fig S4. Zn sensitivity of TpNRAMP3 in yeast.

Expression of *TpNRAMP3* in Zn sensitive strain Δ*zrc1* did not affect the yeast growth when compared with expressing pYES2 in Δ*zrc1*.

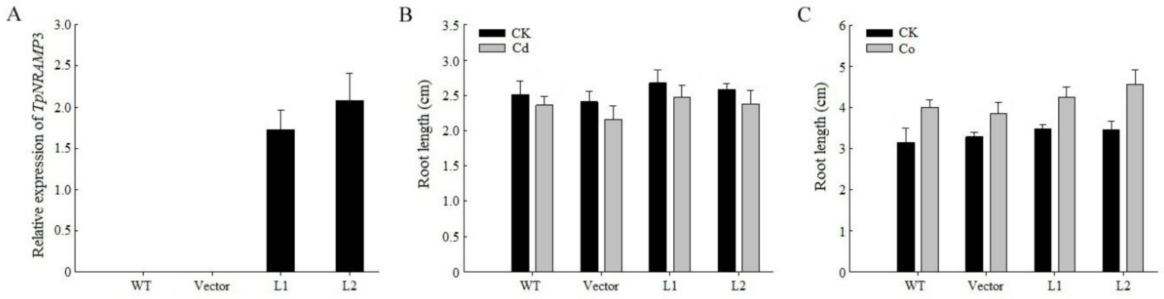

Fig S5. Expression of *TpNRAMP3* in transgenic *Arabidopsis* (A); and root length of transgenic *Arabidopsis* grown on 1/2 MS medium with 25 μM Cd (B) and 80 μM Co (C) stress for 10 days. Value was mean ± standard deviation (three biological replicates).

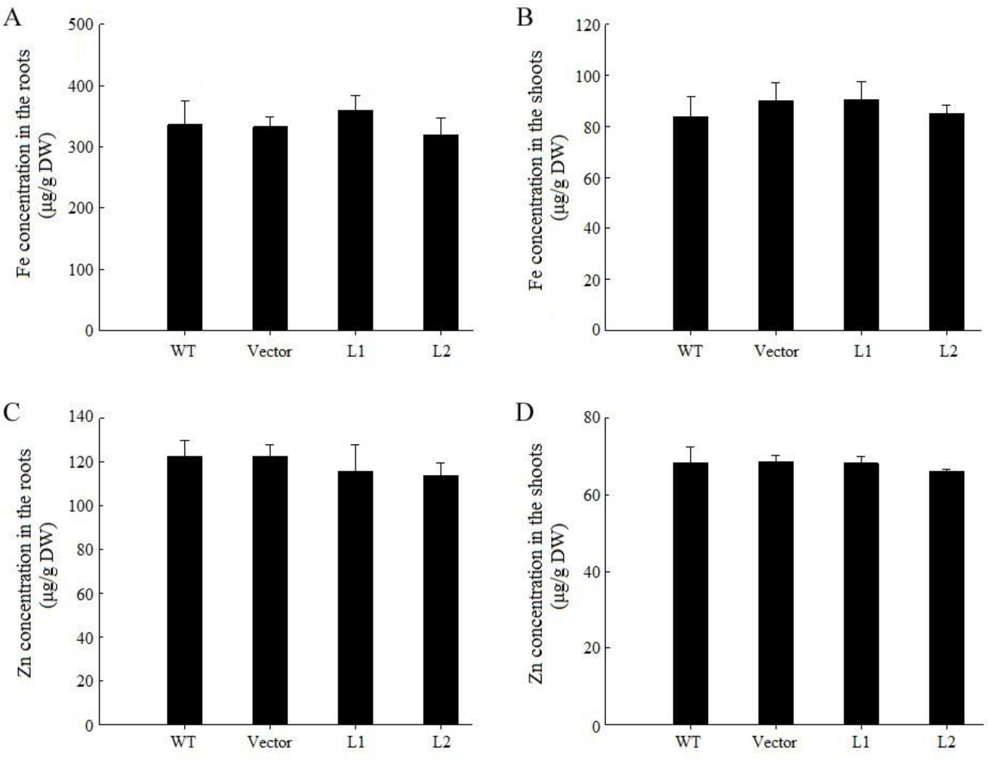

Fig S6. Fe and Zn accumulations in *TpNRAMP5* transgenic *Arabidopsis*.

A-B: Fe concentration in roots (A) and shoots (B); C-D: Zn concentration in roots (C) and shoots (D). Value was mean ± standard deviation (three biological replicates).
